# Siloxane-Based Main-Chain Poly(ionic liquid)s *via* a Debus–Radziszewski Reaction

**DOI:** 10.1021/acspolymersau.1c00029

**Published:** 2021-11-17

**Authors:** Manuel Reiter, Atefeh Khorsand Kheirabad, Miriam M. Unterlass, Jiayin Yuan

**Affiliations:** † Department of Materials and Environmental Chemistry (MMK), Stockholm University, 10691 Stockholm, Sweden; ‡ Institute of Applied Synthetic Chemistry, TU Wien, 1060 Vienna, Austria; § Institute of Materials Chemistry, TU Wien, 1060 Vienna, Austria

**Keywords:** poly(ionic liquid), glass transition temperature, polysiloxane, Radziszewski reaction

## Abstract

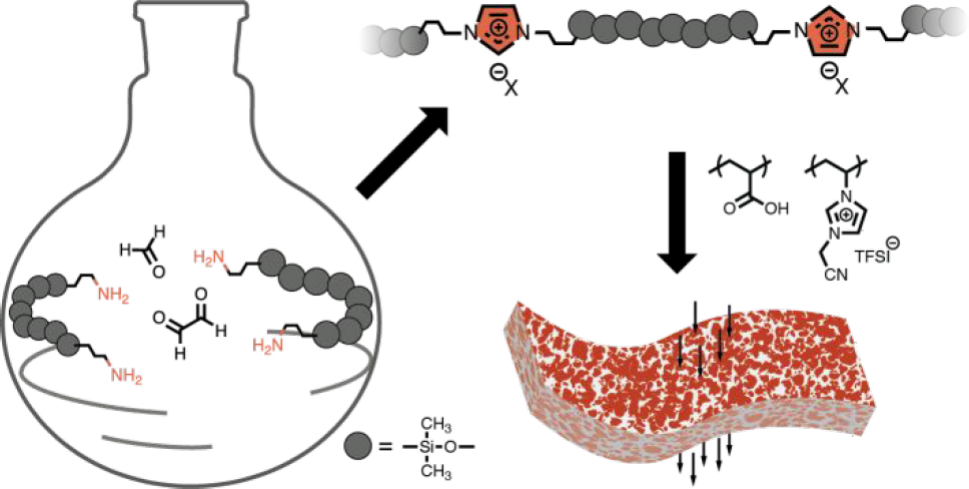

Herein, we synthesized a series of siloxane-based poly(ionic liquid)s (PILs) with imidazolium-type species in the main chain *via* the multicomponent Debus–Radziszewski reaction. We employed oligodimethylsiloxane diamine precursors to integrate flexible spacers in the polymer backbone and ultimately succeeded in obtaining main-chain PILs with low glass transition temperatures (*T*_g_*s*) in the range of −40 to −18 °C. Such PILs were combined with conventional hydrophobic vinylimidazolium-based PILs for the fabrication of porous membranes *via* interpolyelectrolyte complexation with poly(acrylic acid), which leads to enhanced mechanical performance in the tensile testing measurements. This study will enrich the structure library of main-chain PILs and open up more opportunities for potential industrial applications of porous imidazolium-based membranes.

## Introduction

Poly(ionic liquid)s (PILs) have recently served as a substantial class of functional polymers that carry ionic liquid (IL)-like species in the repeating unit. PILs exhibit widely tunable (electro)chemical and thermomechanical properties, which make them attractive for applications particularly in quasi-solid-state electrolytes, porous membranes, actuators, and sensors.^1−5^ Synthetically, numerous routes have been developed to obtain these materials either by chain- and step-growth polymerizations of IL monomers or postpolymerization modifications of available polymer chains. If the ionic species are directly incorporated into the polymer backbone, such polymers are described as main-chain PILs. Typically, they are prepared in a stepwise fashion, *e.g*., *via* a Menshutkin-type reaction^6,7^ or click chemistry.^8,9^ A judicious choice of the precursor molecules allows the favored repeating unit to be structured by tuning both the charged species and the nonionic fragments separating them. The chemical nature and the length of the latter, the so-called spacers, strongly affect the thermal and (electro)chemical properties of the resultant materials.^10−12^ Recently, the introduction of flexible siloxane units to the polymer backbones was found to promote segmental motion of the chains and lead to low *T*_g_s and high ionic conductivity in side-chain PILs, where charged species are located in the side groups of the polymer.^13−17^ In the essence of this discovery, it would be of great interest to apply such flexible siloxane spacers also in their main-chain analogues. Among the various classes of PILs, imidazolium-type ion pairs have been studied the most due to their easy accessibility and favorable properties when employed in PILs, such as high polarity as well as chemical and thermal stability.^18−20^ Main-chain PILs are commonly synthesized from mono/bis-substituted imidazole precursors.^6,21,22^ Recently, the Debus–Radziszewski reaction was found to be efficient in preparing imidazolium-based main-chain PILs under mild and industrially viable conditions.^23^ Although some spacer designs including aliphatic/aromatic fragments and ethylene oxide units were introduced, the great versatility of this approach is yet needed to be further expanded.

Meanwhile, the utilization of PILs in porous polyelectrolyte membranes has gained considerable interest, as they combine various ionic species with porous structures and give rise to unique properties and unusual transport mechanisms.^24^ In this context, our group has previously developed a method to fabricate freestanding porous membranes *via* an interpolyelectrolyte complexation mechanism between a hydrophobic PIL and a weak organic multiacid such as commercial poly(acrylic acid) (PAA).^25^ This methodology was further applied to prepare porous membranes based on a main-chain PIL with a −C_4_H_8_– aliphatic spacer, which was synthesized *via* the Debus–Radziszewski reaction.^26^ However, these porous membranes suffered from low mechanical performance that has limited their industrial use.

In this contribution, we set out to expand the scope of the Debus–Radziszewski reaction toward siloxane-based main-chain PILs. By using oligodimethylsiloxane diamine precursors under aqueous and mild conditions, we prepared 1,3-disubstituted imidazolium-type PILs with unusually low *T*_g_s. Moreover, we studied two essential reaction parameters and their influence on the structure and properties of the resultant PILs. Finally, we fabricated composite porous membranes from a mixture of such “liquid-like” main-chain PIL with a common hydrophobic vinylimidazolium-type PIL and PAA. The flexibility of the siloxane spacers explicitly improved the mechanical properties of these porous membranes.

## Results and Discussion

**Scheme 1:**
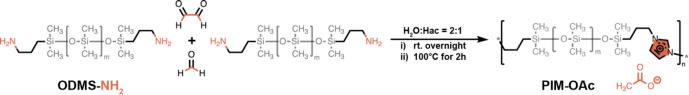
Debus–Radziszewski Reaction toward an Imidazolium-Based Main-Chain Polysiloxane

For the preparation of siloxane-based main-chain PILs with imidazolium-type IL species in every repeating unit of the polymer backbone, we used an amine-terminated dimethylsiloxane oligomer ODMS-NH_2_ of a low molecular weight (MW) as a starting material for the Debus–Radziszewski reaction (MW of 900–1000 g mol^–1^ as stated by the supplier, corresponding to 10–11 dimethylsiloxane units). Its size exclusion chromatography (SEC) trace is given in Figure S1A. Glyoxal and formaldehyde were used as carbonyl compounds (Scheme 1), thereby forming a polymer backbone containing 1,3-disubstituted imidazolium moieties separated by dimethylsiloxane oligomer spacers. Acetate served as the counteranion and was provided by the reaction medium (a mixture of water and acetic acid with a volume ratio fixed at 2:1). Addition of a premixed aqueous solution of carbonyl compounds to a solution of ODMS-NH_2_ was accompanied by an immediately visible increase in viscosity. To facilitate the formation of high-MW polymers and yet enable magnetic stirring for the reaction, a concentration of 40% (mass ratio m/m between monomers and mixture) was chosen. In a recent article, Lindner observed the formation of high-MW polymers with a narrow MW distribution under nonstoichiometric conditions using an excess of carbonyl compounds (molar ratio of amine per carbonyl was 0.7).^23^ Hence, we applied a 1.2-fold excess of carbonyl compounds in a first set of experiments (molar ratio of 0.85).

**Figure 1:**
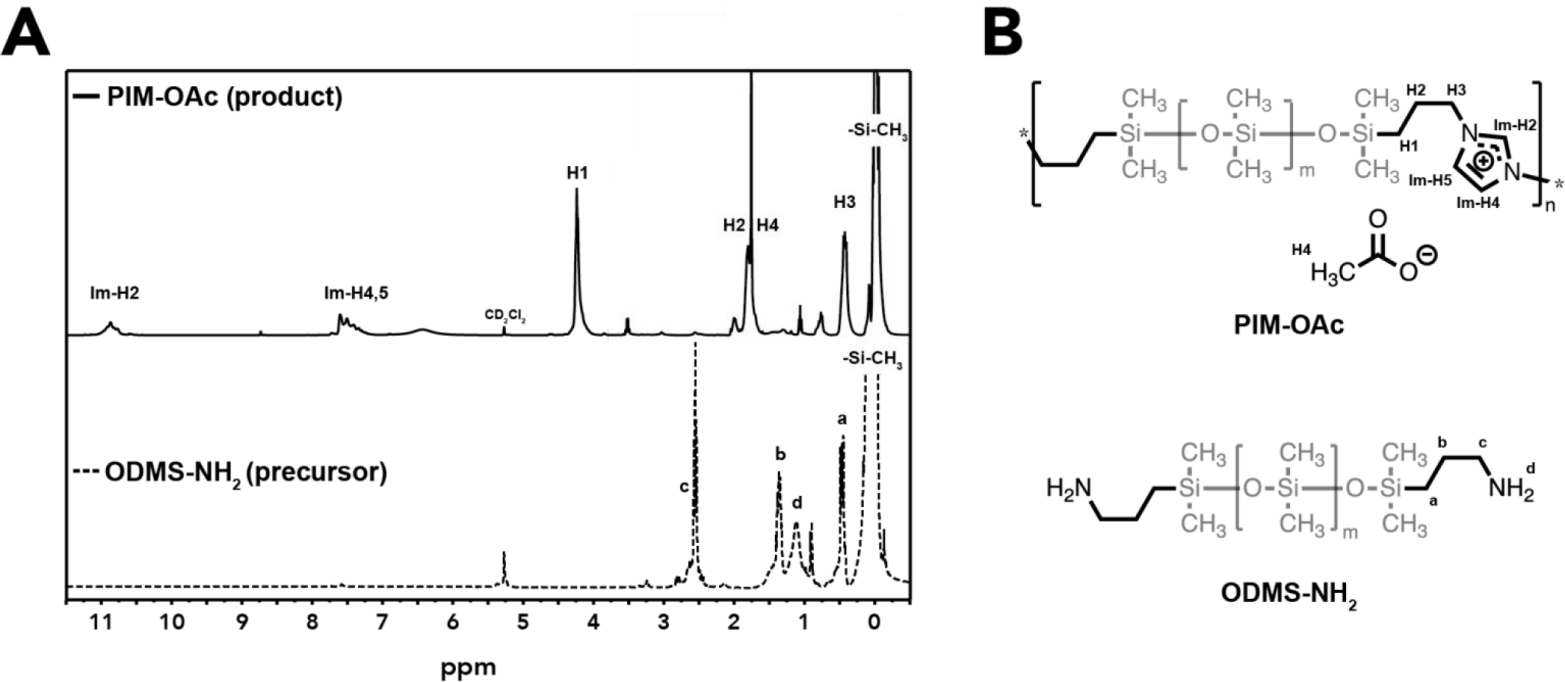
^1^H NMR spectra (A) of product isolated from the reaction mixture and precursor. Labels (B) correspond to peaks in spectra.

The initially clear and colorless reaction mixture turned opaque along the consumption of precursor. We attribute this change to possible impurities in ODMS-NH_2_, which could be of a non- and monoaminated nature, and thus precipitated from the reaction mixture. Evidence is provided by the ^1^H NMR spectrum of the polymer in the water-soluble brownish phase (top spectrum in Figure 1): all characteristic signals for imidazolium moieties are present, in particular Im-H2 and Im-H4,5, which correspond to H-atoms directly attached to the ring. This indicates the formation of fully closed imidazolium rings during polymerization. Peaks related to the propyl segments, which separate oligosiloxane and imidazolium (CH_2_–H1, −H2, and −H3), are observed to shift downfield when compared to the precursor. This was expected when considering the higher electron demand of imidazolium cations over primary amines. Finally, a peak around 0 ppm related to Si–CH_3_ protons confirms the presence of siloxane moieties. Therefore, this clear, brownish phase was identified to contain the product (thereafter referred to as PIM-OAc). The product exhibited a brownish color, which is likely to be caused by a Maillard-type reaction of the precursor diamine and glyoxal.^23,27^ In this context, even a tiny amount of byproduct would cause a strong discoloration, which lets us assume that impurities in our product are negligible (otherwise being entirely black). The spectrum of the second, off-white solid phase that precipitated from solution during polymerization is shown in Figure S2, and mostly PDMS-based chemical shifts can be observed, as demonstrated by a dominant peak around 0 ppm, thus pointing out possible contamination of the starting material by a non- or monoaminated precursor. Interestingly, the integral related to the Si–CH_3_ peaks in the spectrum of PIM-OAc indicates the presence of only 2–3 dimethylsiloxane units under these initially tested reaction conditions. Consequently, the oligosiloxane segments separating imidazolium groups in each repeating unit of the backbone are much shorter than expected from the precursor (10–11 dimethylsiloxane units). This could be either attributed to the contamination that lead to an overestimation of the number of dimethylsiloxane units in the precursor or the rapidly increasing viscosity during the reaction, which could prefer shorter oligomers to be polymerized, leaving longer ones behind. Due to similar solubility of these impurities to ODMS-NH_2_, practically no reasonable purification technique could be applied by us prior to use.

**Figure 2:**
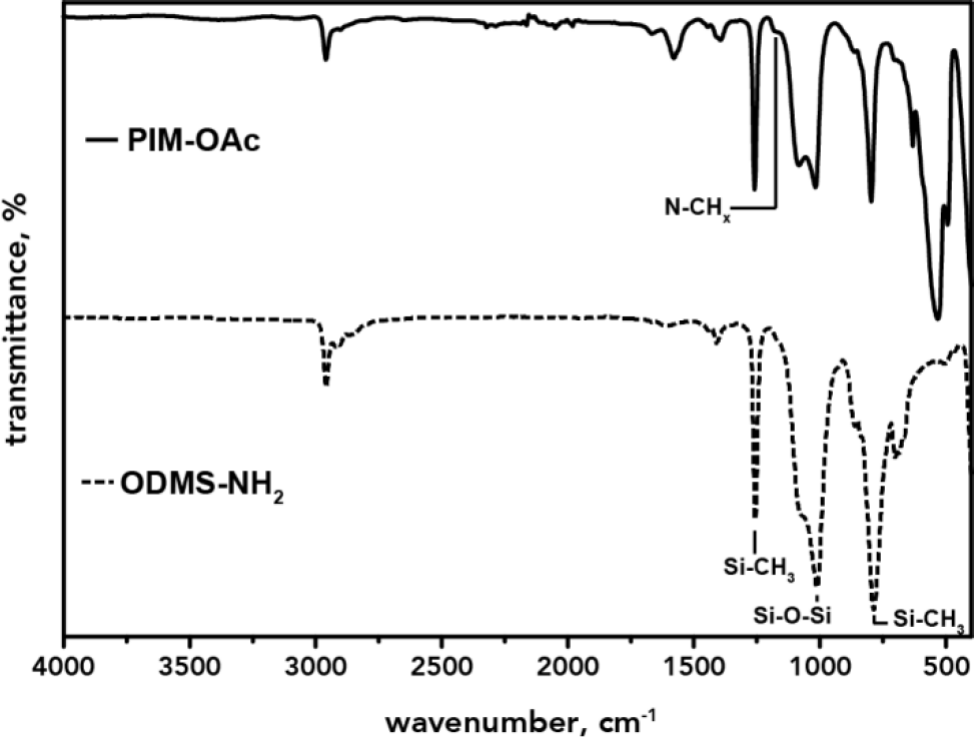
ATR-FTIR spectra of product (top) and precursor (bottom) with essential stretching modes highlighted.

The formation of imidazolium rings was further confirmed by ATR-FTIR spectroscopy. The upper spectrum in Figure 2 shows the product and reveals the combinational vibration of N–CH/N–CH_2_/N–CH_3_ stretching, characteristic for aromatic imidazolium rings, around 1170 cm^–1^.^18,28−30^ Moreover, all essential siloxane-type bands found in the precursor are also present in the product, *i.e*., v(Si–CH_3_) ≈ 790 and 1260 cm^–1^ and v(Si–O–Si) ≈ 1020 cm^–1^.^28^

SEC measurement was performed to confirm the polymeric nature of the product, showing a bimodal MW distribution with a number-average apparent MW of 9500 g mol^–1^ (*M*_w_ = 22 000 g mol^–1^) and a dispersity of 2.32 (Figure S3, determined by refractive index detector against pullulan standards).

**Figure 3:**
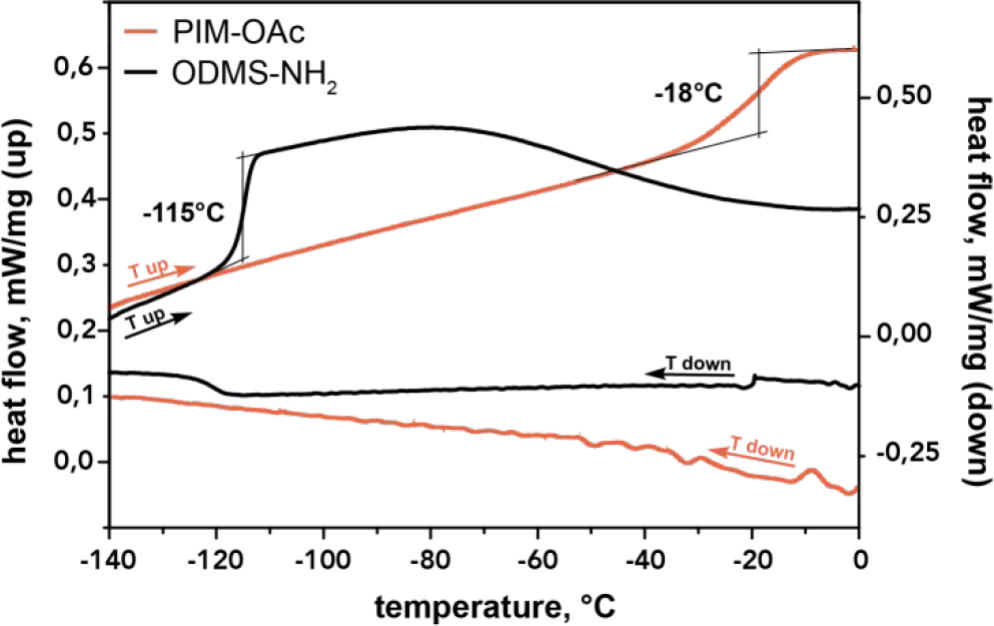
DSC traces of PIM-OAc (orange) and precursor (black). The heating rate was set to 20 °C min^–1^, and the cooling curves were set to 10 °C min^–1^. *T*_g_ was calculated as the local maximum of the first-order derivative of heat flow.

Next, the glass transition temperature (*T*_g_) was determined by differential scanning calorimetry (DSC). Figure 3 shows the heating curve of PIM-OAc and indicates a moderately low glass transition around −18 °C. This is comparable to *T*_g_s reported for aliphatic-carbon-based spacers.^23^ We attribute this moderately low *T*_g_ to the fact that the siloxane segments are short; thus, the properties of PIM-OAc are determined by the joint effect of the oligosiloxane and the ionic species.

To further decrease the *T*_g_, we exchanged acetate anions with weakly coordinating and bulky bis(triuoromethane sulfonyl)imide (TFSI) anions, as they were previously found to be efficient in tuning the thermal properties of siloxane-based PILs.^17^ The anion metathesis was performed by adding an aqueous solution of LiTFSI to a solution of PIM-OAc. Immediately upon addition, the polymer containing the more apolar TFSI anions precipitated out of solution and was isolated *via* centrifugation. The “liquid-like” brownish product was characterized by ^1^H NMR spectroscopy, and indeed, all acetate anions were replaced by TFSI anions. Figure S4A shows the spectra of both polymers before (bottom) and after (top) anion metathesis. The characteristic peak related to acetate (1.8 ppm for the CH_3_ group) is absent in the product, hence confirming quantitative conversion. For both spectra, the Im-H2 peak is not visible due to fast proton exchange with the solvent methanol-*d*_4_. ^13^C NMR spectroscopy is in full accordance with that the top spectrum in Figure S4B reveals the characteristic quartet for TFSI anions (overlaps with imidazolium). In the same time, peaks related to acetate (175 and 20 ppm)^31^ vanish in the product. Another strong indication for the presence of TFSI anions is provided by ATR-FTIR spectroscopy: Figure S4C depicts the spectra of polymers bearing TFSI (top) and acetate (bottom). Clearly, all characteristic stretching modes related to TFSI are observed in the product, *i.e*., v_as_(SO_2_) ≈ 1349 cm^–1^, v_as_(CF_3_) ≈ 1181 cm^–1^, and v_s_(SO_2_) ≈ 1135 cm^–1^.^32,33^

Despite the success of this two-step approach toward a TFSI-containing PIL (termed PIM-TFSI thereafter), several washing steps were rather time-consuming and tedious. Thus, we modified our approach toward direct formation of PIM-TFSI from the polymerization mixture without prior isolation of the other reagents. Therefore, the concentrated polymerization mixture was first diluted with water and after removing unwanted precipitate by decanting off, an aqueous solution of LiTFSI was added to form the PIM-TFSI as an oily precipitate.

**Figure 4:**
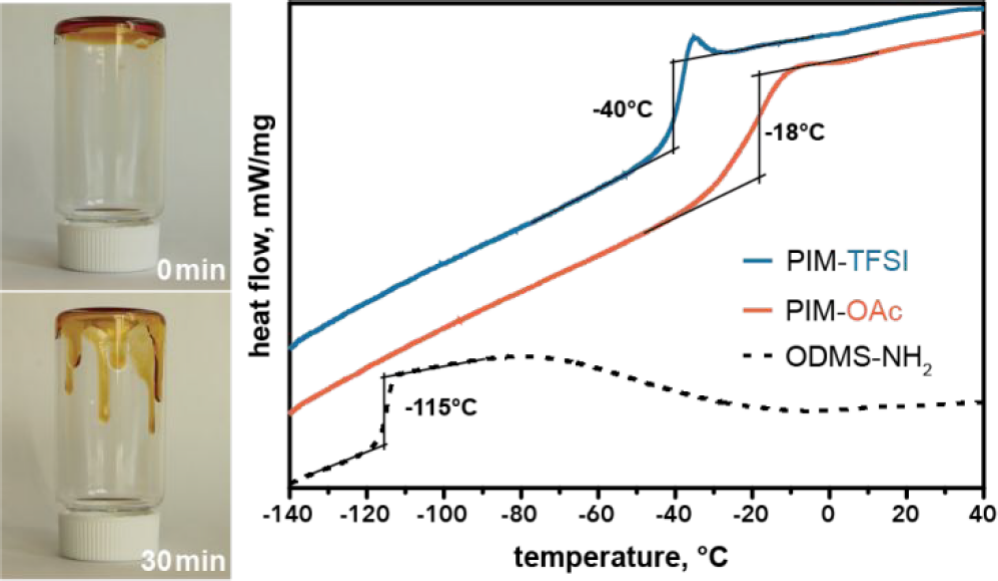
Flow demonstration of PIM-TFSI at room temperature and the DSC heating traces of PIM-TFSI (blue), PIM-OAc (orange), and the precursor (dotted) the precursor.

The flowability of the dried PIM-TFSI in bulk is demonstrated in Figure 4 when the small glass vial is turned upside-down: within 10 min, the brownish polymer starts to flow downward the walls of the glass container due to gravity, and at 80 °C, it only takes about 5 min for the polymer to reach the bottom. This “liquid-like” nature is underpinned by the DSC heating trace shown in Figure 4 revealing a *T*_g_ at −40 °C, ∼22 °C lower than that of the PIM-OAc. This value is among the lowest ever reported for an imidazolium-type main-chain PIL and is comparable to Lindner’s report utilizing aliphatic and PEO-based spacers.^23,26^ Clearly, the exchange of acetate with TFSI anions has a dramatic impact on the thermal properties.

Although the conditions used for the Debus–Radziszewski reaction successfully yielded the imidazolium-containing polysiloxanes PIM-OAc/TFSI, the flexible segments separating the charged species were found to be unexpectedly short, only 2–3 dimethylsiloxane units. Consequently, the ionic part of the polymer mostly determines its thermal properties, and the siloxane-based spacers contribute comparably less, thus leading to only a moderately low *T*_g_ at −18 °C. As mentioned earlier, we attributed the short length of the siloxane segments to either contamination and/or rapidly increasing viscosity that favors shorter ODMS-NH_2_ to polymerize. To cast a deeper view, we carried out a new set of experiments varying the concentration of the reaction mixture. We hypothesized that diluting the system should increase the relative chance of the longer oligomers to react, and consequently, the average length of the oligodimethylsiloxane spacer in the repeating unit would increase. This would also allow the thermal properties of the PIL to be tuned. The initially high concentration of 40% (amine + carbonyls/mixture mass ratio) was lowered to 33, 2, and 1%, while the carbonyl compounds were kept in a 1.2-fold excess to amine (amine/carbonyl molar ratio of 0.85). Polymerization and anion metathesis were carried out under the same conditions as before, and all experiments resulted in brownish but clear, “liquid-like” polymers (termed PIM-TFSI-*x*% thereafter, *x*% denotes the concentration).

The structures were confirmed by ^1^H NMR and ATR-FTIR spectroscopy (Figure S5A,B). Figure S5C compares the integrals related to the Si–CH_3_ peaks in the corresponding ^1^H NMR spectra for polymers prepared at different concentrations. Clearly, the values increase from 15.8 to 58.7 with decreasing concentrations, which translate into approximately 3 to 10 dimethylsiloxane units per repeating unit, respectively. Figure S5D illustrates the calculated spacer lengths, showing the longest siloxane segment (*ca*. 10 units) being obtained under the most dilute conditions (PIM-TFSI-1%). This strongly supports our hypothesis that a high monomer content facilitates polymerization of preferably shorter oligomers, and dilution of the mixtures allows also longer precursor oligomers to participate in the polymerization. As a result, longer precursor molecules can join the backbone and increase the average length of the repeating unit. This is also indicated by an increasing yield of polymerization (wt% polymer per total reactants) under dilute conditions: PIM-TFSI-40% was obtained in a relatively low yield of 20 wt %, whereas the polymerization of PIM-TFSI-2% was nearly quantitative (96 wt %). However, further dilution of the system to 1% presumably hampers the polymerization, as the yield dropped to 75 wt % for PIM-TFSI-1%, indicating the complex role of the concentration in the Debus–Radziszewski reaction.

We were curious if the concentration of the reaction mixture also affects the length of the resulting polymers, especially since longer siloxane segments inevitably increase the size of the repeating unit. In addition to the SEC trace of PIM-OAc, a polymer that contains only 2–3 dimethylsiloxane units and shows a number-average molecular weight (*M*_n_) of 9500 g mol^–1^ (Figure S3), we analyzed a polymer with 10 dimethylsiloxane units, *i.e*., PIM-OAc-1%. Figure S6 shows the MW distribution with an *M*_n_ of 57 700 g mol^–1^ (*M*_w_ = 196 800 g mol^–1^). Although both MW values are the apparent *M*_n_s, both polymers were measured under the same SEC conditions and thus are comparable. This indicates that much longer chains are formed under more dilute conditions.

**Figure 5:**
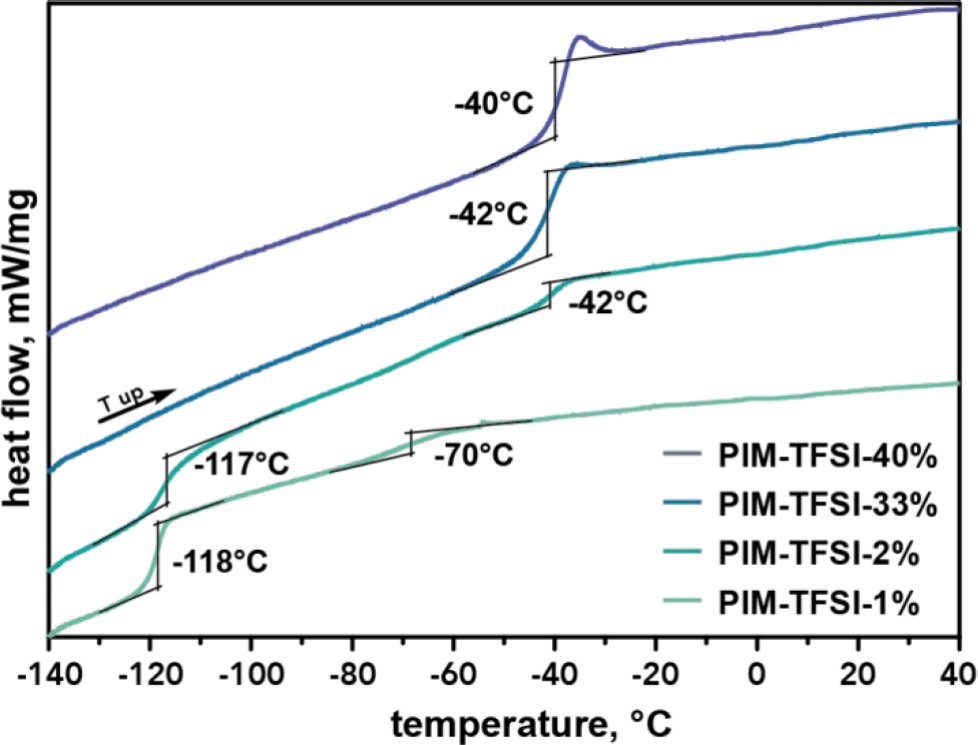
DSC heating traces for decreasing the monomer concentration in the polymerization from top to bottom.

By modifying the length of the flexible spacer between 3 and 10 units, we were also able to tune the *T*_g_. Figure 5 shows the DSC heating traces with decreasing concentration from top to bottom. PIM-TFSI-33% revealed a *T*_g_ at −42 °C, which is similar to PIM-TFSI-40% (top trace). Surprisingly, this glass transition becomes less prominent for lower concentrations as in PIM-TFSI-2%. Simultaneously, another glass transition was observed at −117 °C, which is similar to the *T*_g_ of the precursor at −115 °C. Two different *T*_g_s were already observed for polysiloxanes with imidazolium-type IL species in the side chain.^17^ This phenomenon appears only for longer spacers, which strongly indicates that microphase separation of the ionic and apolar segments is promoted by the extension of the oligosiloxane spacer. For PILs with short siloxane spacers (PIM-TFSI-40% and PIM-TFSI-33%), the ionic component seems to dominate the thermal properties, and consequently, only one glass transition is observed. When the flexible oligosiloxane segments increase to a certain level, as in PIM-TFSI-2%, both the apolar and ionic parts seem to contribute equally to the polymer’s thermal properties. This would also explain the small “bump” that is observed around −70 °C, which is likely to be a superposition of both individual glass transitions, a phenomenon widely known to occur for polymer blends^34^ and block copolymers.^35,36^ For even lower concentrations, as in PIM-TFSI-1%, the second *T*_g_ becomes the major phase transition. To exclude the possibility of low-MW impurities causing this phenomenon, especially the diamine precursor molecules and siloxane-based cycles, we performed electrospray ionization (ESI) mass spectrometry in the low-MW region of ODMS-NH_2_, PIM-TFSI-40%, and PIM-TFSI-1%. As shown in Figures S7–9, none of the aforementioned species were found in both polymers

Since we were able to tune the spacer length by varying the concentration of the polymerization mixture, we wondered if the stoichiometry, as another crucial parameter, could play a similar role. As already mentioned, a slight excess of carbonyl compounds (molar ratio of 0.85) was chosen to facilitate the formation of high-MW compounds.^23^ This resulted in the formation of “liquid-like” PILs with an apparent number-average MW of 9500 g mol^–1^. To further explore the impact of stoichiometry, we performed two experiments with an even higher excess of carbonyls, *i.e*., a molar ratio of 0.43 and 0.11, and one with an excess of diamine (molar ratio of 1.7). All reactions were carried out in a similar fashion to previous polymerizations and at a concentration of 2%, as PIM-TFSI-2% was found to contain the longest siloxane-based spacer of all (*ca*. 10 units).

All polymerizations delivered a brownish polymeric phase (termed PIM-TFSI-*y* thereafter, *y* denotes the amine/carbonyl molar ratio); however, the ones resulting from a high excess of carbonyls (PIM-TFSI-0.43 and PIM-TFSI-0.11) were solid instead of “liquid-like”. These PILs also showed very limited solubility in common organic solvents (see Figure S10), which made it impossible to perform common solution NMR or SEC measurements. However, the formation of imidazolium rings was confirmed by ATR-FTIR spectroscopy as shown in Figure S11A. We assume that these PILs either feature cross-links or show too high of an MW due to the dramatic imbalance in the monomer ratio. By contrast, the polymerization performed with an excess of diamine (PIM-TFSI-1.7) yielded a “liquid-like”, soluble PIL that is similar to previous experiments at a molar ratio of 0.85. Although ^1^H NMR spectroscopy (Figure S11B) confirmed the formation of imidazolium rings for PIM-TFSI-1.7, additional peaks suggest contamination with precursor molecules. These impurities remained in the product even after thorough water-washing steps, indicating that they are “dissolved” in the product polymer chains. The ATR-FTIR spectrum (Figure S11A, bottom spectrum) also confirms the formation of imidazolium species.

**Figure 6:**
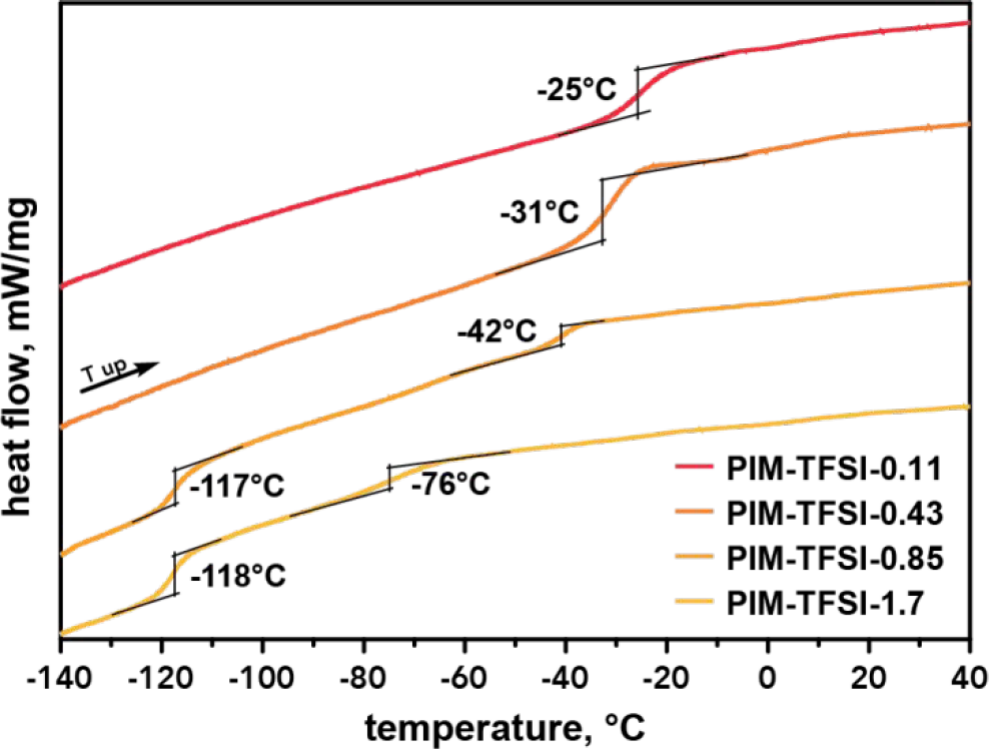
DSC heating traces for PILs prepared at decreasing carbonyl content from 0.11 to 1.7.

Figure 6 presents the DSC heating traces from top to bottom with increasing molar ratio from 0.11 to 1.7 and, as expected from the PILs’ appearance, a declining *T*_g_. The PIL obtained at the highest carbonyl content (PIM-TFSI-0.11) exhibits a glass transition at −25 °C (top curve), which is surprisingly high in comparison to the one obtained at a molar ratio of 0.85 (*T*_g_ = −40 °C). A slightly lower molar ratio (PIM-TFSI-0.43) also results in a lower *T*_g_ of −31 °C. Further decreasing the carbonyl content yet keeping an excess (molar ratio of 0.85) reveals a second glass transition at −117 °C, as seen previously. PIM-TFSI-0.85 therefore fits the decline of *T*_g_ and marks as an important point in the transition from solid to “liquid-like” PILs. Further reduction of carbonyls (causing excess of diamine) again shows two phase transitions.

**Figure 7:**
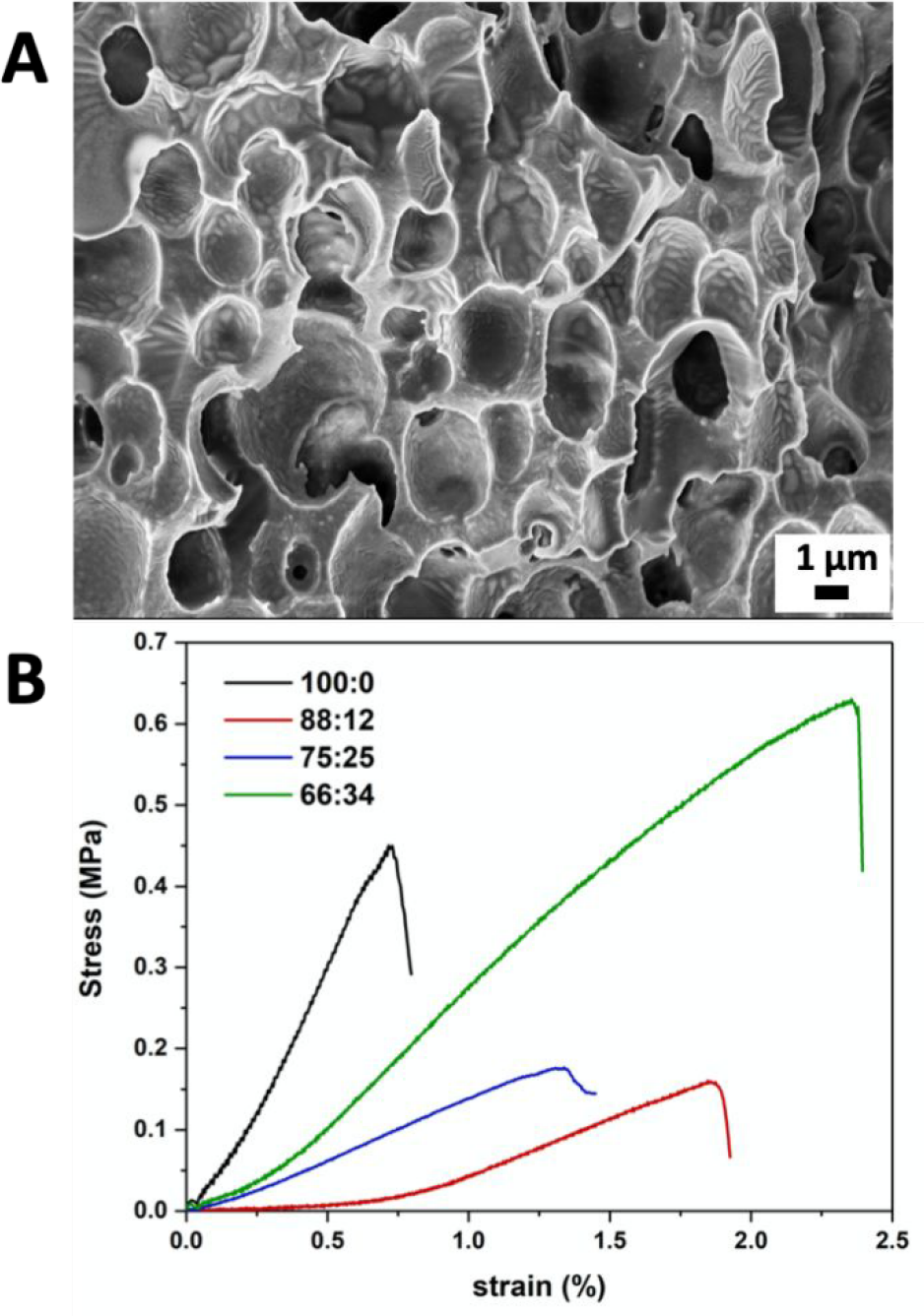
(A) Cross-sectional SEM images of the hybrid porous membrane with a relative wt.% ratio of PCMVIm-TFSI/PIM-TFSI-40% = 66:34 and (B) tensile stress–strain plot of hybrid porous membranes with different relative wt.% ratios of PCMVImTFSI:PIM-TFSI-40%.

As mentioned earlier, the introduction of a flexible oligodimethylsiloxane spacer into the main-chain PIL might improve the mechanical performance of the target porous membranes. In following with the interpolyelectrolyte complexation method between a hydrophobic PIL and an organic weak multiacid that our group established recently,^25^ a series of new composite porous membranes were prepared for this study. These membranes paired PAA (*M*_w_ = 10^5^ g/mol) with a mixture of two different PILs, *i.e*., poly[1-cyanomethyl-3-vinylimidazolium bis(trifluoromethane sulfonyl)imide] (termed PCMVIm-TFSI, which was previously used by us in the membrane fabrication, see Figure S12) and PIM-TFSI-40% (with the lowest number of siloxane units). The synthetic procedure and structural characterization of PCMVIm-TFSI, the fabrication procedure of porous membranes, and the relative amount of each of the two PILs in the composite membranes are provided in the Supporting Information. As shown in a representative scanning electron microscopy (SEM) image of the composite membrane in Figure 7A, a porous membrane was successfully formed from a PIL mixture of 66 wt % of PCMVIm-TFSI and 34 wt % of PIM-TFSI-40%. Cross-sectional SEM images and the pore size distribution histograms of these membranes are provided in Figures S13 and S14, respectively. The mechanical properties of the composite membranes with various compositions of the PIL mixture were tested in the dry state and compared with the PCMVIm-TFSI-PAA membrane without PIM-TFSI-40%. As shown in Figure 7B, the PCMVIm-TFSI-PAA membrane breaks at a lower strain of 0.75% compared to 1.2–2.5% for the composite membranes. However, the relative amount of PIM-TFSI-40% in the porous membrane has a complex impact on the tensile strength at failure. The replacement of 34% of PCMVIm-TFSI with PIM-TFSI-40% enhanced the tensile strength from 0.45 MPa (a membrane free of PIM-TFSI-40%) to 0.62 MPa. While decreasing the PIM-TFSI-40% amount in the PIL mixture will decrease the tensile strength, a higher PIM-TFSI-40% amount, *e.g*., 50 wt %, failed to produce an intact membrane for mechanical tests. This interesting phenomenon is indicative of a comprehensive role of PIM-TFSI-40% in the composite membranes that is of our future research interest.

## Conclusion

In summary, for the first time, oligosiloxane-based diamines as precursor were utilized in a Debus–Radziszewski reaction to yield siloxane-based imidazolium-type PILs, expanding the scope of this industrially relevant multicomponent reaction. Viscous, “liquid-like” PILs in bulk were obtained and further modified by anion metathesis to reach a *T*_g_ as low as −40 °C. We found unexpectedly short siloxane-based segments separating the imidazolium units in the polymer backbone, which prompted us to optimize two crucial reaction parameters: concentration and stoichiometry. Both seem to have dramatic effects on the resultant PILs, and particularly, the concentration could be used to tune the length of the spacers between 3 to 10 dimethylsiloxane units. We assumed that a more dilute system facilitates the reaction of longer precursor molecules. This is accompanied by a dramatic change of thermal properties: PILs with short spacers exhibit only one phase transition at −42 °C, whereas PILs with longer ones reveal a second *T*_g_ at −117 °C. The latter became more prominent for longer spacers, and simultaneously, the imidazolium-related *T*_g_ diminished. The stoichiometry was found to change the physical state of PIL products. A higher excess of carbonyls leads to solid polymers with a relatively high *T*_g_ of −25 °C, while lowering the carbonyl content, yet applying formaldehyde and glyoxal in excess, gave rise to “liquid-like” products again, thereby confirming the “nonstoichiometric” nature of the Debus–Radziszewski reaction. Finally, porous membranes containing the ODMS-based main-chain PILs showed much improved mechanical properties.

## Supporting Information Available

Detailed methods, materials, and synthesis as well as NMR spectra, ATR-FTIR spectra, ESI mass spectra, and SEC traces (PDF)

## Abbreviations

PIL: poly(ionic liquid)
ODMS-NH_2_: oligodimethylsiloxane diamine precursor
PIM-OAc: imidazolium-containing product of the Debus–Radziszewski reaction with acetate as a counteranion
PIM-TFSI: PIL resulting from anion metathesis with bis(trifluoromethane sulfonyl)imide counteranions
PCMVIm-TFSI: poly[1-cyanomethyl-3-vinylimidazolium bis(trifluoromethane sulfonyl)imide]
