# Supplementary material for: Siloxane-Based Main-Chain Poly(ionic liquid)s via a Debus–Radziszewski Reaction
Source: ACS Polym Au. 2021 Nov 17;2(2):80–7. doi: 10.1021/acspolymersau.1c00029 (PMC9011398; doi:10.1021/acspolymersau.1c00029)
Supplement: Supplementary file 1 — lg1c00029_si_001.pdf [file lg1c00029_si_001.pdf]

Supporting information for

Siloxane-based main-chain poly(ionic liquid)s *via* Debus-Radziszewski reaction

Manuel Reiter[1,2,3†], Atefeh Khorsand Kheirabad[1], Miriam M. Unterlass[2,3†*], Jiayin Yuan[1*]

[1]Department of Materials and Environmental Chemistry (MMK), Stockholm University, 10691 Stockholm, Sweden
[2]Institute of Applied Synthetic Chemistry, TU Wien, 1060 Vienna, Austria
[3]Institute of Materials Chemistry, TU Wien, 1060 Vienna, Austria
†Manuel Reiter, Laboratory of Polymeric Materials, Department of Materials, ETH Zurich, 8093 Zurich, Switzerland
†Miriam M. Unterlass, Department of Chemistry, University of Konstanz, 78457 Konstanz, Germany

General Methods:

^{1}H- and ^{13}C-solution nuclear magnetic resonance (NMR) spectra were recorded at room temperature on a Bruker DPX-400 spectrometer operating at 400/101 MHz equipped with a BBI and BBO probe, respectively. Dichloromethane-d_2, chloroform-d_3, methanol-d_4 and DMSO-d_6 were used as solvents for all measurements. Spectra were analyzed using MestReNova.

Attenuated total reflection Fourier transform infrared (ATR-FTIR) spectroscopy was conducted on a Varian 610-IR FT-IR spectrometer equipped with a Specac Goldengate single reflection ATR accessory and a diamond element. A deuterated triglycine sulfate (DTGS) room temperature detector was used and data analysis was performed with Resolution Pro by Agilent. Resolution was set to 4 cm^{-1} and spectra were recorded from 4000 to 400 cm^{-1}.

Differential scanning calorimetry (DSC) tests were performed on a Netzsch DSC 214 Polyma equipped with a CC300 liquid N_2 cooling device. Measurements were carried out at a cooling rate of 10°C/min and various heating rates (5, 10, 20, 40 and 250°C/min), in a temperature range of -160 to 100°C and under a constant nitrogen flow rate of 40 mL/min. Samples were placed in closed, cold-weldable 40 µL Concavus Al crucibles.

Aqueous size-exclusion chromatography (SEC) measurements were conducted on a PSS Novema XL Max analytical linear 10 µm column (calibration with pullulan), with a PSS Novema S VS as pre-column (calibration with dextran). A mixture of 80% aqueous acetate buffer (pH = 4.5) and 20% methanol was used as eluent at a flow rate of 1.0 mL/min. Organic solvent SEC was performed on a PSS SDV 5 µm column with a porosity of 1000, 100000 and 1000000 Å (calibrated with PMMA) with a PSS SDV 5 µm as pre-column. THF was used as eluent at a flow rate of 1.0 mL/min. For both aqueous and organic solvent SEC a PSS SECurity[2] refractive index detector and a TSO UV-1000 UV/VIS-detector were used. The samples were measured at 25°C and an injection volume of 100 µL.

The morphology of the samples was characterized by scanning electron microscopy (SEM) conducted on a JEOL 7000 operated at 3 kV. Samples were coated with a thin gold layer for 60 seconds before examination.

Mechanical tensile tests were conducted on an Instron 5,960 universal testing machine (Instron, MA, USA). All the specimens for mechanical testing were cut into rectangular strips with a width of 3 mm and a length of 30 mm, by a razor blade. The thickness of the specimens was confirmed by measuring the cross-section of the stripped specimens. Tensile strengths and failure strains were recorded when the fracture occurred. The Young's moduli were calculated from the slopes of the linear regions of the stress-strain curves and fracture toughnesses were calculated by integrating the areas under the stress-strain curves. All the reported data were the average of over three to five strips of the same samples.

Electrospray ionization mass spectroscopy (ESI-MS): An Agilent's Jet Stream ESI interface was used in positive ion mode (ESI^+) with the following settings: capillary voltage 3 kV, nebulizer pressure 20 psi, sheath gas flow rate 11 L/min, sheath gas temperature 250°C, dry gas temperature 250°C, and dry gas flow rate 14 L/min. Spectra were collected from m/z 50 to 3000 Da. The injection volume was 1 µL. All samples were dissolved in Methanol with concentration of 1 mg/ml.

Chemicals:

The aminopropyl-terminated oligodimethylsiloxane precursor (ODMS-NH_2) was purchased from Gelest and used as received. The precursor has a molar mass of 900-1000 g mol^{-1}, as stated in the product description. The apparent molar mass of M_w = 1 700 g mol^{-1} was determined by SEC measurement in THF (RI detector).
Formaldehyde (37% in water, Fisher Scientific), glyoxale (40% in water, Alfa Aesar), acetic acid (glacial, VWR), 1-vinylimidazole (99%, Acros Organics), bromoacetonitrile (TCI Europe) and poly(acrylic acid) (PAA) (M_w 10^5 Da, Sigma Aldrich) were used as received. Spectra/Por 2 Trial Kit with a molecular weight cut-off (MWCO) of 12 - 14 kDa was used for dialysis. Lithium bis(trifluoromethane sulfonyl)imide (LiTFSI, 99%, io-li-tec) was used as received. Solvents, such as methanol (99.9%, Honeywell), ethanol (99.5%, VWR), dimethyl sulfoxide and tetrahydrofuran were used without further purification.

Synthesis:

Preliminary set-up:

In a typical experiment, 1.0 eq. of ODMS-NH_2 (equals two amino groups) was weighed into a three-necked round-bottom flask equipped with a reflux condenser and dissolved in a 1:2 (volume ratio)-mixture of glacial acetic acid and water. The resulting clear solution was cooled down using an ice bath. In a separate flask 0.85 eq. of glyoxale (40 wt.% in water) and formaldehyde (37 wt.% in water) were mixed together and subsequently added dropwise to the ice-cold solution of diamine. Cooling was continued for another 10 min. during which the viscosity increased. The clear solution was allowed to come to room temperature and stirred for 24 h. For reactions performed at higher concentration it was necessary to add more solvent during polymerization to enable magnetic stirring (as viscosity increases). The resulting opaque mixture was refluxed for 2 h and finally concentrated at 80°C under reduced pressure to yield a brownish viscous phase. The product was taken up in 40 mL ethanol (not fully soluble), vortexed and centrifuged at 9000 rpm for 30 min. The remaining "solid-like" phase, which was identified later as an impurity, was washed thoroughly with ethanol and all supernatants were combined and

concentrated. To further purify the crude product it was dialyzed against ethanol for 2 d. The clear brown solution was concentrated again and finally dried at 80°C under high vacuum (10^{-3} mbar) for 24 h to give a dark-brown and flowable polymer.

Table S1: Formulation for Debus-Radziszewski reactions. $n_{diamine}$ refers to a MW of 900-1000 g mol^{-1} (stated by the supplier); molar ratio = $n_{diamine}/n_{carbonyls}$; concentration = $m_{total\ monomer}$ / $m_{mixture}$.

compound	n (mmol)	eq.	m (g)
**ODMS-NH_2**	**5.00**	**2.0**	**5.004**
**glyoxal, 40%**	**5.91**	**1.2**	**0.857**
**formaldehyde, 37%**	**5.93**	**1.2**	**0.481**

molar ratio was kept at 0.85, concentration at 40 wt.%; resulting product PIM-AcO was dialyzed against ethanol (MWCO 12 - 14 kDa).
Yield = 1.238 g of a dark-brownish viscous polymer with an apparent number-average molecular weight M_n of 9,500 g mol^{-1}, according to aqueous SEC measurement (RI detector, see Figure S3 for SEC trace).

^{1}H NMR (CD_2Cl_2, 400 MHz, δ): 10.90 (m, 0.87H, Im-H2), 7.46 (m, 1.76H, Im-H4,5), 4.28 (t, J=6.60 Hz, 4.05H, CH_2-H3), 1.86 (s, 2.74H, CH_2-H2), 1.82 (s, 2.96H, CH_3-acetate), 0.49 (t, 4.00H, CH_2-H1), 0.05 (m, 16.17H, Si-CH_3). ^{1}H NMR (methanol-d_4, 400 MHz, δ): 7.68 (s, 1.39H, Im-H4,5), 4.21 (t, J=6.60 Hz, 4.00H, CH_2-H3), 1.90 (m, 6.06H, CH_2-H2+CH_3-acetate), 0.53 (t, 4.61H, CH_2-H1), 0.10 (m, 16.33H, Si-CH_3).

Anion exchange:

This procedure was only applied for the initial experiment. For further studies (influence of molar ratio and concentration), please refer to the modified set-up below. In a typical experiment 1.2 eq. LiTFSI was dissolved in water and added to an aqueous solution of 1 eq. of PIM-OAc. The corresponding TFSI-containing polymer precipitated immediately from solution and was collected *via* centrifuge at 9000 rpm for 30 min. To remove excess LiTFSI the freshly formed polymer was thoroughly washed with water (suspend, vortex, centrifuge). The remaining polymer was taken up in methanol, concentrated and dried at 80°C under high vacuum (10^{-3} mbar) for 24 h to yield a brownish flowable product. The appearance is similar to the acetate-containing polymer, but its flow is dramatically enhanced.

Table S2: Anion metathesis of Debus-Radziszewski product. $n_{PIM\text{-}OAc}$ refers to an apparent MW of 9 500 g mol^{-1} (SEC).

compound	n (mmol)	eq.	m (g)
**PIM-OAc**	**2.848$^{ref.\ to\ OAc}$**	**1.0**	**1.108**
**LiTFSI**	**3.497**	**1.2**	**1.004**

yield = 1.002 g (57.7wt% of theory), refers to an apparent MW of 14 900 g mol^{-1} (based on the apparent MW of PIM-OAc) and a number of PMDS units per repeating unit of 2 to 3. The product was of dark-brownish appearance and “liquid-like” nature.

^{1}H NMR (methanol-d_4, 400 MHz, δ): 7.61 (s, 1.35H, Im-H4,5), 4.20 (t, J=6.95 Hz, 3.59H, CH_2-H3), 1.89 (m, 4.00H, CH_2-H2), 0.52 (t, J=8.59 Hz, 4.02H, CH_2-H1), 0.08 (m, 14.37H, Si-CH_3). ^{13}C NMR (methanol-d_4, 101 MHz, δ): 123.7 - 119.6 (m, Im-C4,5 + -CF_3), 53.5 (s, CH_2-C3), 25.6 (CH_2-C2), 15.5 (s, CH_2-C1), 0.5 (m, Si-CH_3).

Modified set-up:

The previous set-up was modified to directly give the TFSI-bearing polymers and thus simplify purification. The Debus-Radziszewski reaction was performed according to the procedure above. However, after concentrating the reaction mixture the resulting brownish phase was dissolved in 40 mL of water and the pH changed from acidic to slightly basic by adding ammonia solution (3 wt% in water). After centrifuging at 9000 rpm for 1 h the supernatant was collected and the remaining solid was washed thoroughly with water. The combined supernatants were filtered and concentrated (not fully) at 80°C under reduced pressure. Subsequently, an aqueous solution of 1.2 eq. LiTFSI was added directly to the concentrated solution, which resulted in immediate precipitation of PIM-TFSI-40%. After the product was collected (*via* centrifuge) and washed several times with water it was finally dried at 80°C under high vacuum (10^{-3} mbar) for 24 h.

Study on the effect of concentration:

Table S3: Formulations for polymerizations with different concentrations using the modified work-up procedure. $n_{diamine}$ refers to a MW of 900-1000 g mol^{-1} ; molar ratio for all experiments was kept at 0.85.

concentration	compound	n (mmol)	eq.	m (g)
	ODMS-NH_2	5.00	2.0	5.004
40	glyoxal, 40%	5.91	1.2	0.857
	formaldehyde, 37%	5.93	1.2	0.481
	ODMS-NH_2	2.00	2.0	2.00
33	glyoxal, 40%	2.35	1.2	0.341
	formaldehyde, 37%	2.48	1.2	0.201
	ODMS-NH_2	1.99	2.0	1.99
2.0	glyoxal, 40%	2.35	1.2	0.341
	formaldehyde, 37%	2.40	1.2	0.195
	ODMS-NH_2	4.99	2.0	4.99
1.0	glyoxal, 40%	5.93	1.2	0.860
	formaldehyde, 37%	5.91	1.2	0.480

PIM-TFSI-40%: yield = 1.002 g (=20 wt%); ^{1}H NMR (methanol-d_4, 400 MHz, δ): 7.61 (s, 1.31H, Im-H4,5), 4.20 (t, J=6.95 Hz, 3.73H, CH_2-H3), 1.89 (m, 4.00H, CH_2-H2), 0.52 (t, J =8.59 Hz, 4.11H, CH_2-H1), 0.08 (m, 15.76H, Si-CH_3).

PIM-TFSI-30 %: yield = 0.794 g (=40 wt%); ^{1}H NMR (methanol-d_4, 400 MHz, δ): 7.62 (s, 1.87H, Im-H4,5), 4.21 (t, J=6.88 Hz, 3.84H, CH_2-H3), 1.89 (m, 4.00H, CH_2-H2), 0.54 (m, 5.21H, CH_2-H1), 0.10 (m, 29.55H, Si-CH_3).

PIM-TFSI-2.0 %: yield = 1.914 g (=96 wt%); ^{1}H NMR (methanol-d_4, 400 MHz, δ): 7.64 (s, 1.50H, Im-H4,5), 4.21 (s, 3.91H, CH_2-H3), 1.92 (s, 4.00H, CH_2-H2), 0.55 (s, 4.02, CH_2-H1), 0.11 (s, 48.43, Si-CH_3).

PIM-TFSI-1.0 %: yield = 3.752 g (=75 wt%); ^{1}H NMR (methanol-d_4, 400 MHz, δ): 7.63 (s, 1.59H, Im-H4,5), 4.84 (starting material), 4.21 (s, 4.00H, CH_2-H3), 2.92 (starting material), 1.91 (s, 4.04H, CH_2-H2), 1.69 (starting material), 0.54 (s, 5.79H, CH_2-H1), 0.10 (s, 58.71H, Si-CH_3).

Study on the effect of molar ratio:

Table S4: Formulations for polymerizations with different molar ratio using the modified work-up procedure; $n_{diamine}$ refers to a MW of 900-1000 g mol^{-1}; concentration was kept at 2.0% for all experiments.

molar ratio	compound	n (mmol)	eq.	m (g)
	**ODMS-NH_2**	**2.50**	**2.0**	**2.50**
**0.11**	**glyoxal, 40%**	**23.68**	**9.5**	**3.436**
	**formaldehyde, 37%**	**23.89**	**9.6**	**1.939**
	**ODMS-NH_2**	**5.04**	**2.0**	**5.04**
**0.43**	**glyoxal, 40%**	**11.93**	**2.4**	**1.731**
	**formaldehyde, 37%**	**11.68**	**2.3**	**0.948**
	**ODMS-NH_2**	**1.99**	**2.0**	**1.99**
**0.84**	**glyoxal, 40%**	**2.35**	**1.2**	**0.341**
	**formaldehyde, 37%**	**2.40**	**1.2**	**0.195**
	**ODMS-NH_2**	**2.51**	**2.0**	**2.51**
**1.7**	**glyoxal, 40%**	**1.50**	**0.6**	**0.217**
	**formaldehyde, 37%**	**1.52**	**0.6**	**0.123**

PIM-TFSI-0.11: yield = 1.116 g (=44 wt%); ^{1}H NMR: missing due to insolubility of polymer.

PIM-TFSI-0.43: yield = 1.105 g (=22 wt%); ^{1}H NMR: missing due to insolubility of polymer.

PIM-TFSI-0.84: yield = 1.914 g (=96 wt%); ^{1}H NMR (methanol-d_4, 400 MHz, δ): 7.64 (s, 1.50H, Im-H4,5), 4.21 (s, 3.91 H, CH_2-H3), 1.92 (s, 4.00H, CH_2-H2), 0.55 (s, 4.02, CH_2-H1), 0.11 (s, 48.43, Si-CH_3).

PIM-TFSI-1.7: yield = 0.760 g (=30 wt%); ^{1}H NMR (methanol-d_4, 400 MHz, δ): 7.64 (s, 1.42H, Im-H4,5), 4.21 (t, J=7.05 Hz, 4.00H, CH_2-H3), 2.85 (starting material), 1.91 (m, 3.61H, CH_2-H2), 1.65 (starting material), 0.55 (t, J=8.60 Hz, 5.00H, CH_2-H1), 0.10 (m, 91.42H, Si-CH_3).

Figures:

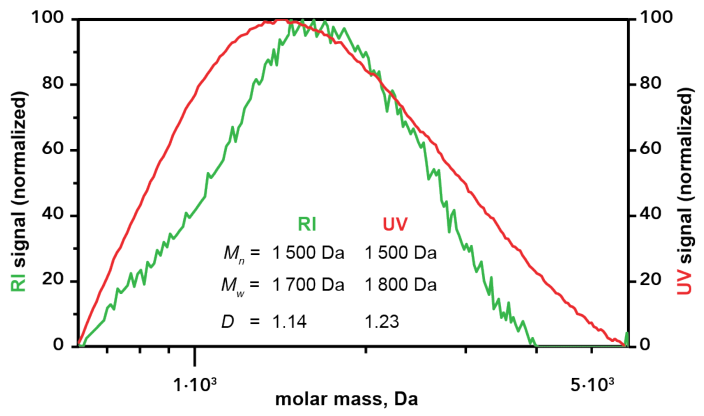

Figure S1: SEC trace of the precursor ODMS-NH_2.

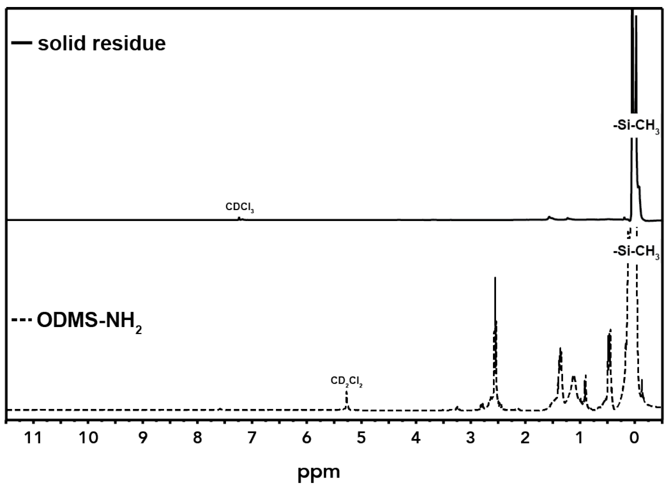

Figure S2: ^{1}H-NMR spectra of the solid residue from polymerization (top) and the precursor (B).

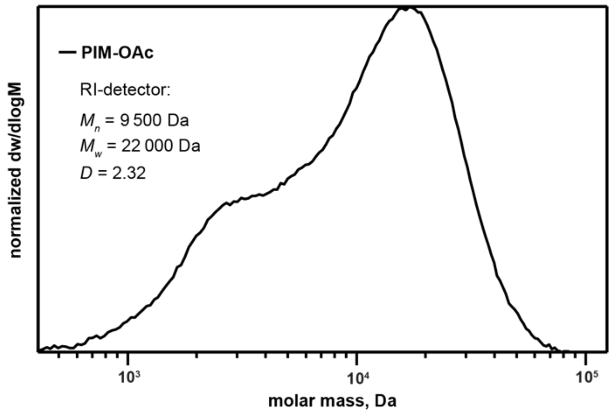

Figure S3: MW distribution of PIM-OAc obtained from aqueous SEC and determined with RI detector against pullulan standards.

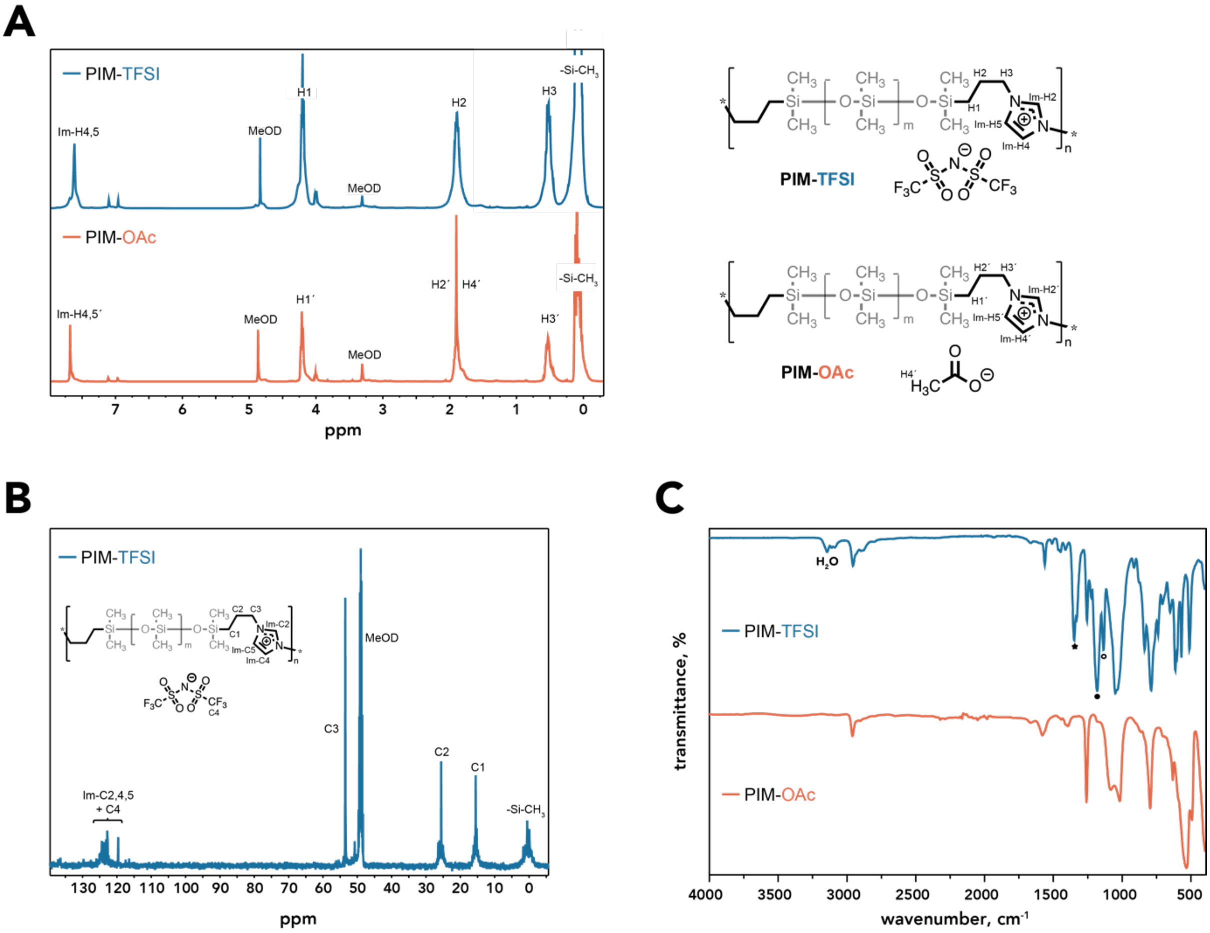

Figure S4: (A) ^{1}H-NMR spectra of the TFSI-bearing polymer (top, after anion exchange) and the acetate-bearing polymer (bottom, prior to exchange); both spectra were measured in methanol-d$_4$; chemical structures are given for better understanding. (B) the corresponding ^{13}C-NMR spectrum of PIM-TFSI measured in methanol-d$_4$. (C) ATR-FTIR spectra of both polymers.

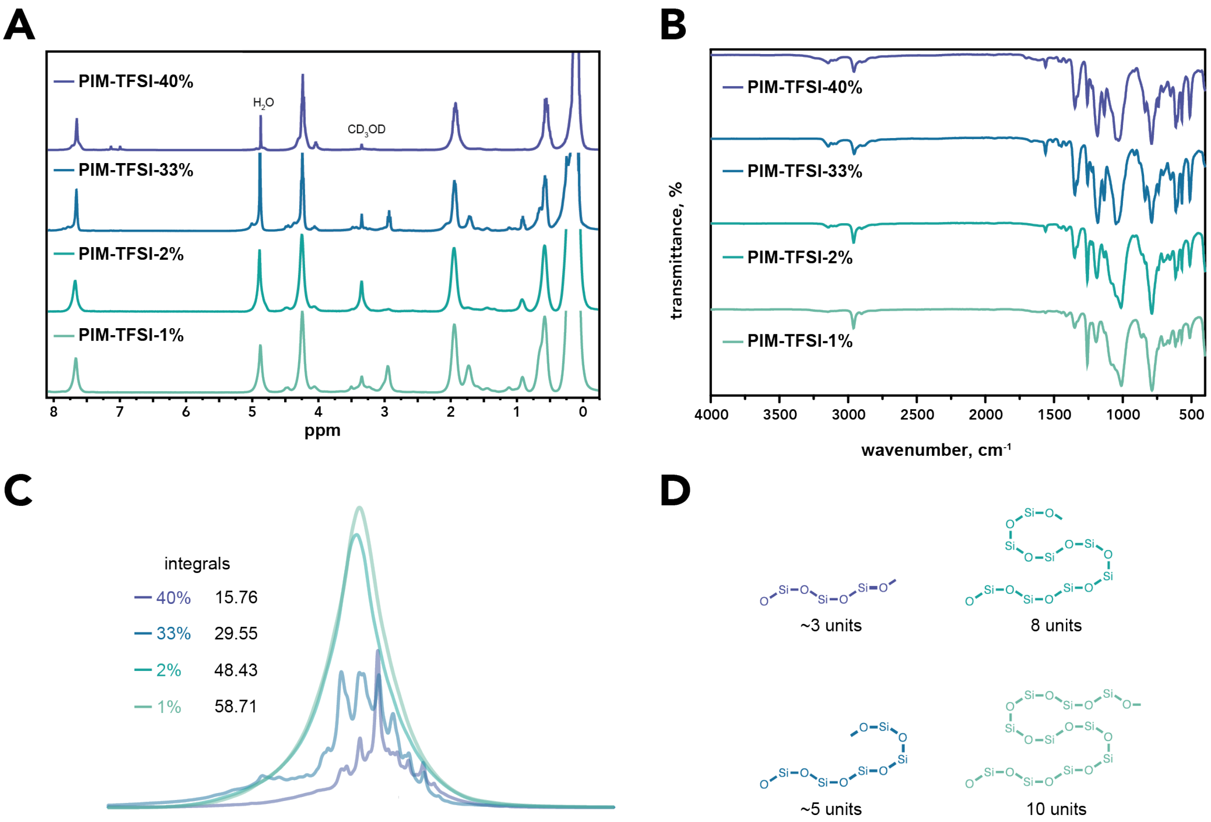

Figure S5: (A) ^{1}H-NMR spectra of Debus-Radziszewski reaction products PIM-TFSI-x% (x% denotes the concentration) with a decreasing concentration from top to bottom. (B) the corresponding ATR-FTIR spectra. (C) highlights the Si-CH_3 peaks in the ^{1}H-NMR spectra above; integrals were calculated relative to CH_2-groups of the propyl-segments, which were set to 4. (D) Chain-lengths based on these integrals represent the numbers of siloxane units in the repeating units of the polymers.

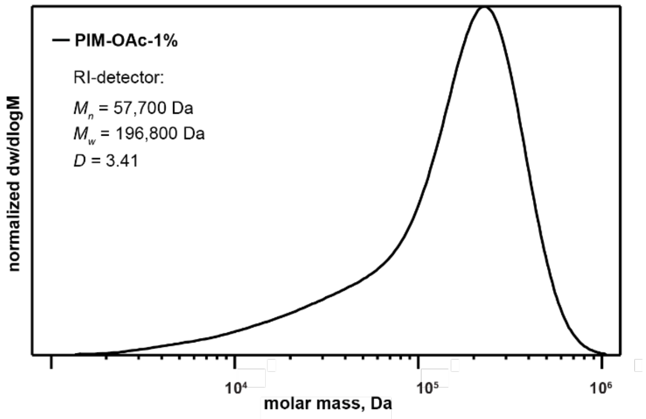

Figure S6: MW distribution of PIM-OAc-1% obtained from aqueous SEC and determined with RI detector against pullulan standards.

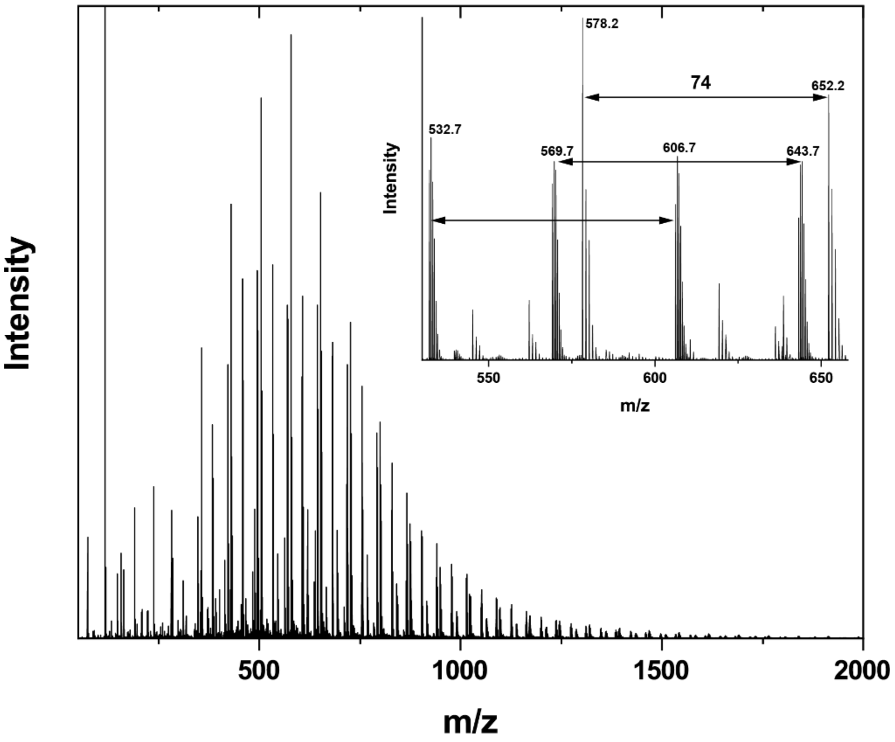

Figure S7: ESI mass spectrum of ODMS-NH_2.

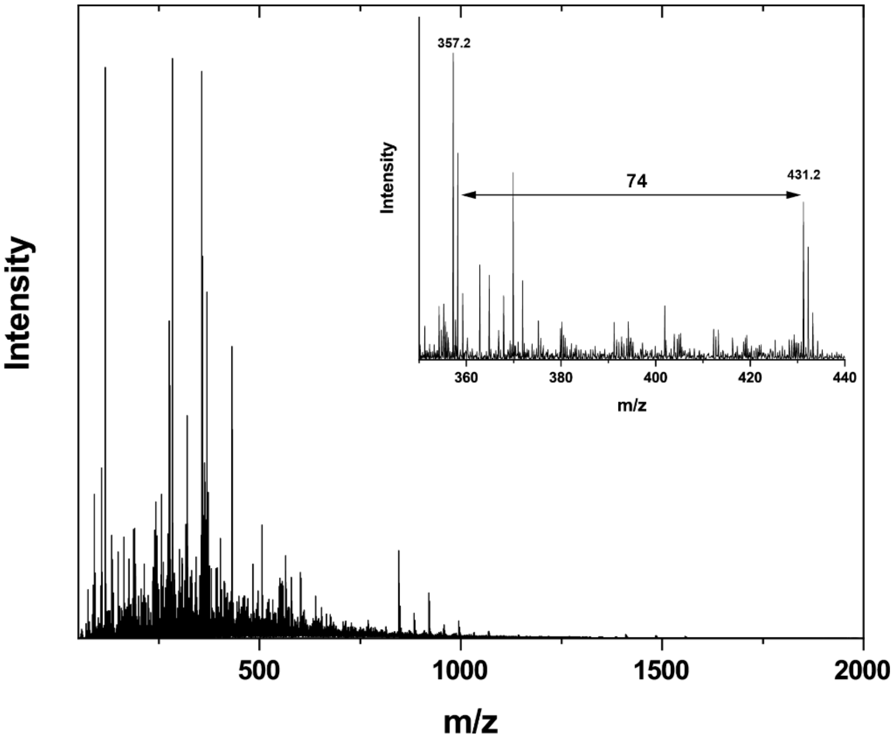

Figure S8: ESI mass spectrum of PIM-TFSI-40%.

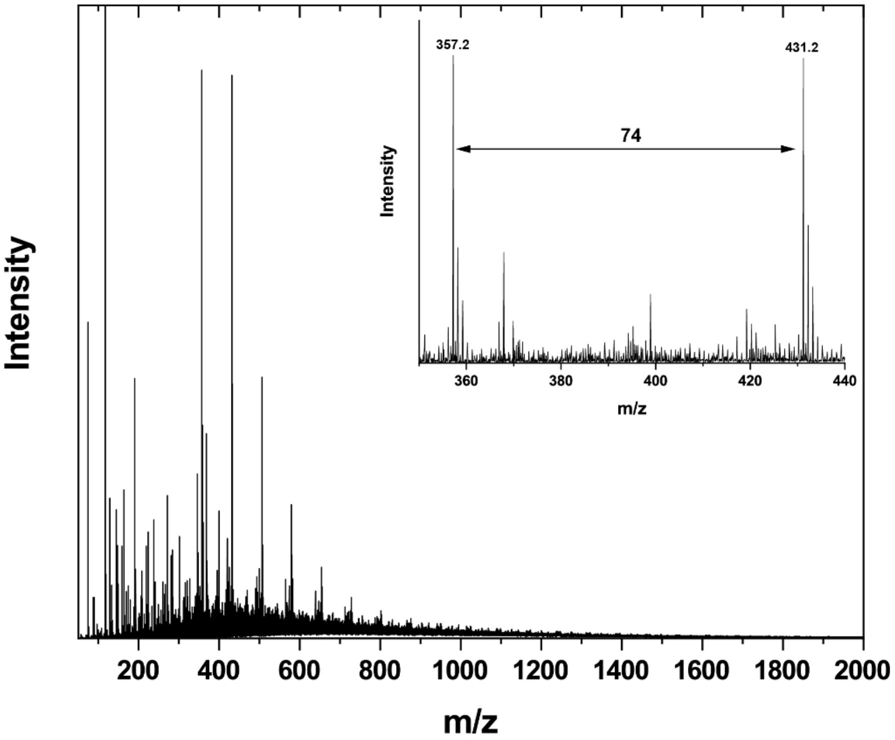

Figure S9: ESI mass spectrum of PIM-TFSI-1%.

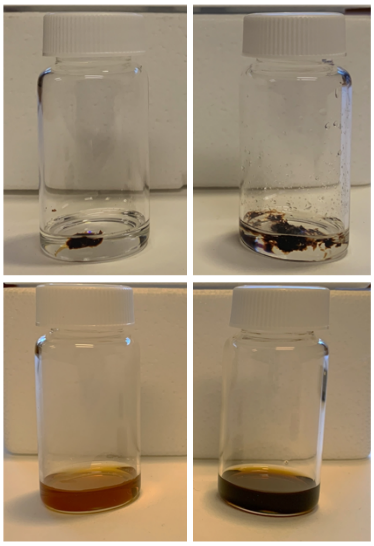

Figure S10: Photograph of partially dissolved "solid-like" samples after treatment in DMSO at 80°C overnight. Left: PIM-TFSI-0.43, right: PIM-TFSI-0.11

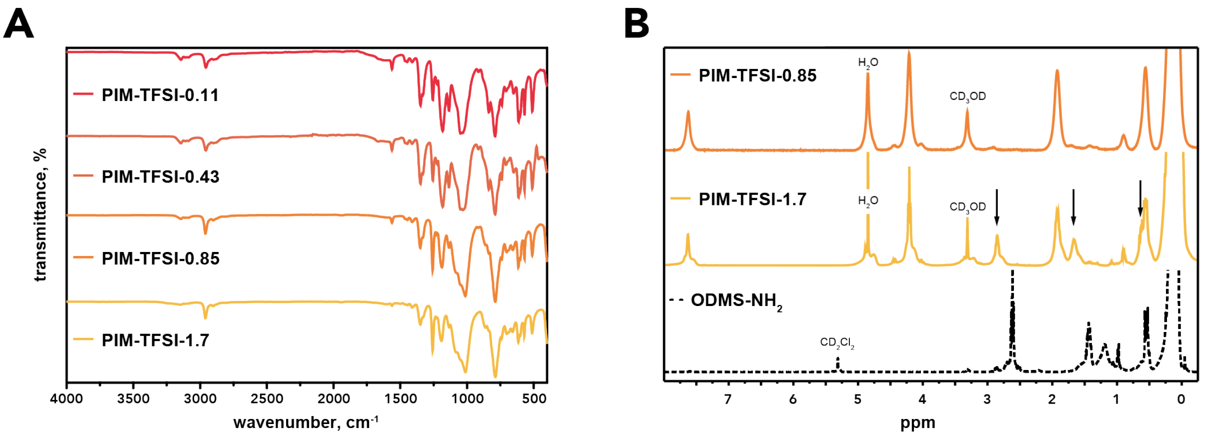

Figure S11: ATR-FTIR (A) and ^{1}H-NMR (B) spectra of PIM-TFSI-y (y denotes the amine/carbonyl molar ratio) prepared with decreasing carbonyl content from top to bottom. Arrows indicate impurities; 0.11 and 0.43 are not depicted due to limited solubility.

Fabrication procedure of porous membranes:

Synthesis of 3-cyanomethyl-1-vinylimidazolium bromide monomer (CMVImBr) and poly(CMVImBr) (PCMVImTFSI)

1-Vinylimidazole (10 g) and bromoacetonitrile (15 g) were dissolved in 125 ml diethyl ether. It is to be noted there should not be any direct mixture of 1-vinylimidazole and bromoacetonitrile. The reaction mixture was stirred at room temperature for 12 h. The obtained precipitate was filtered off, washed with diethyl ether three times and dried under high vacuum at 50°C for 12 h. CMVImBr monomer (10 g) and AIBN initiator (80 mg) were dissolved in degassed DMSO (100 ml) under nitrogen. The reaction mixture was stirred, heated to and kept at 60°C for 12 h under nitrogen atmosphere. Afterwards, the solution was dropped into excessive amounts of THF (1 L). The obtained precipitate was collected by filtration, washed with THF three times and dried under high vacuum at 50°C overnight. PCMVImBr (10 g) was dissolved in deionized water (200 ml). A 100 ml of bis(trifluoromethylsulfonyl)imide lithium salt (12 g) solution was added to the PCMVImBr aqueous solution. After addition, the reaction mixture was allowed to stir for 2 h, then the precipitate was filtered off, washed several times with deionized water, and dried under high vacuum at 70 °C for overnight.

Fabrication of porous composite PIL/PAA membranes:

200 mg of PCMVImTFSI and 100 mg of PIM-TFSI-40% (amounts calculated based on a relative wt% of PCMVImTFSI), and (36 + 12) mg of PAA were dissolved homogenously in 2 ml of DMSO to give a 1:1 molar ratio mixture of imidazolium and carboxylate units. The solution was then poured onto a glass plate and dried at 80°C for 2 h. The obtained sticky film was soaked in 0.2 wt% aqueous ammonia solution. After 2 h of soaking, a free-standing membrane was detached easily from its underlying glass substrate and washed with pure water. It was finally dried in air until constant weight.

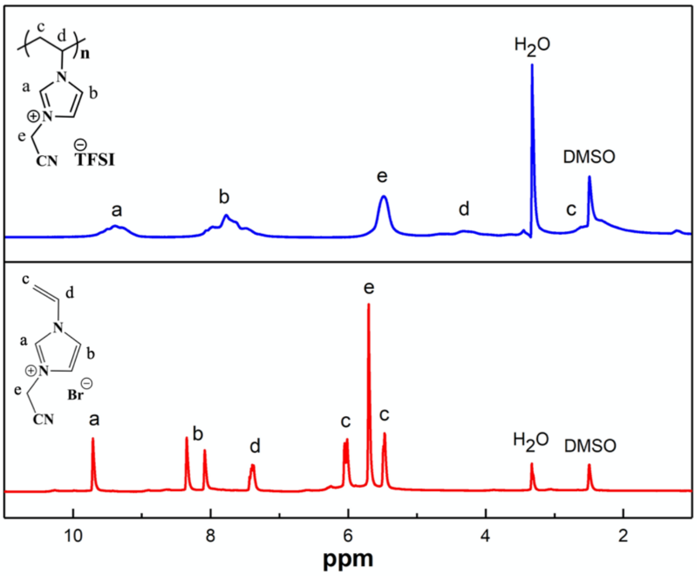

Figure S12: ^{1}H-NMR spectra of CMVImBr monomer and PCMVImTFSI in DMSO-d_6.

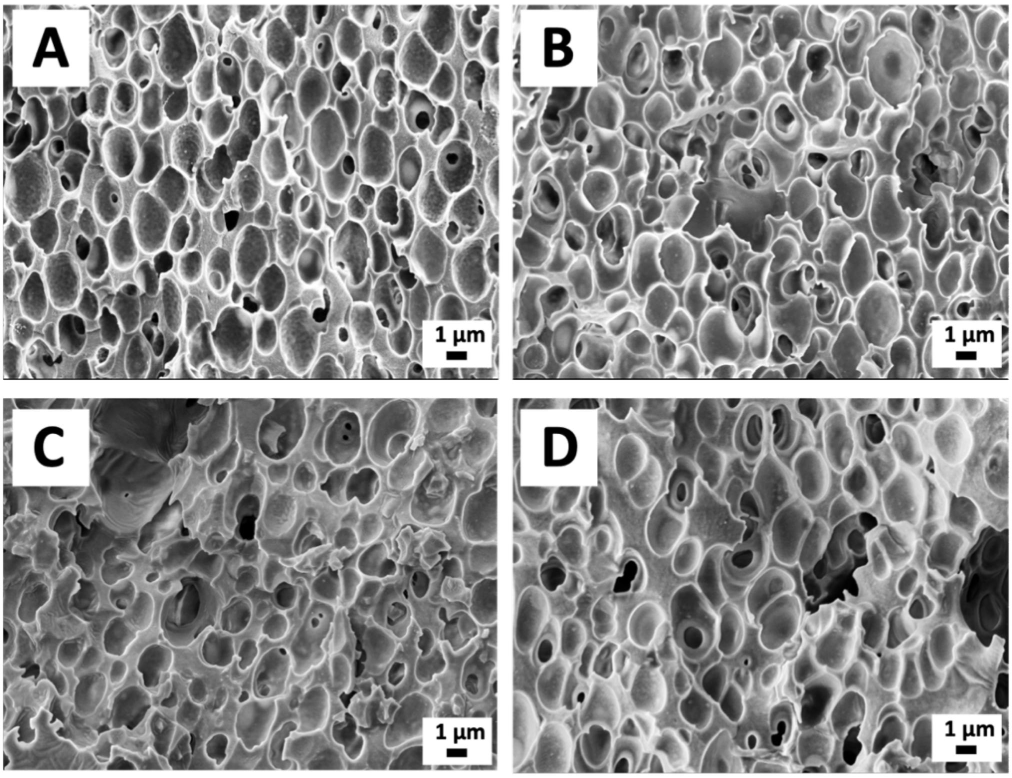

Figure S13: Cross-sectional SEM images of the composite porous membranes with different relative wt% ratio of PCMVImTFSI:PIM-TFSI-40%: (A) 100:0, (B) 88:12, (C) 75:25, (D) 66:34.

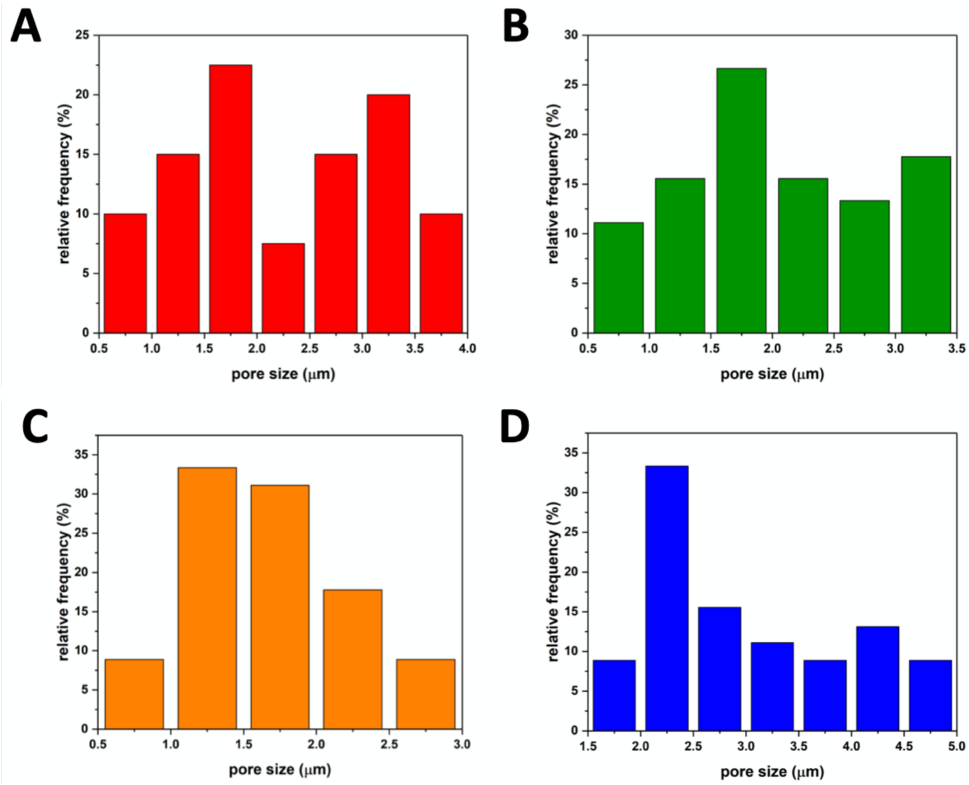

Figure S14: The pore size distribution histograms of the composite porous membranes with different relative wt% ratio of PCMVImTFSI:PIM-TFSI-40%: (A) 100:0, (B) 88:12, (C) 75:25, (D) 66:34. Average pore sizes are: 2.3±0.9 µm, 2.3±0.9 µm, 1.7±0.6 µm, 3.1±1.0 µm, respectively.
